# The Effectiveness of Virtual Reality–Based Training on Cognitive, Social, and Physical Functioning in High-Functioning Older Adults (CoSoPhy FX): 2-Arm, Parallel-Group Randomized Controlled Trial

**DOI:** 10.2196/53261

**Published:** 2024-06-05

**Authors:** Ewa Szczepocka, Łukasz Mokros, Jakub Kazmierski, Karina Nowakowska, Anna Łucka, Anna Antoszczyk, Javier Oltra-Cucarella, Walter Werzowa, Martin Moum Hellevik, Stavros Skouras, Karsten Bagger

**Affiliations:** 1 Department of Old Age Psychiatry and Psychotic Disorders Medical University of Lodz Lodz Poland; 2 Department of Clinical Pharmacology Medical University of Lodz Lodz Poland; 3 Senopi AG (Aktiengesellschaft) Zurich Switzerland; 4 Department of Health Psychology University Miguel Hernández de Elche Elche Spain; 5 The Music Medicine Consultancy Vienna Austria; 6 Stiftelsen CatoSenteret Son Norway; 7 Department of Biological and Medical Psychology University of Bergen Bergen Norway

**Keywords:** cognitive functions, head-mounted-display, healthy seniors, virtual reality, well-being, mobile phone

## Abstract

**Background:**

Virtual reality (VR) has emerged as a promising technology for enhancing the health care of older individuals, particularly in the domains of cognition, physical activity, and social engagement. However, existing VR products and services have limited availability and affordability; hence, there is a need for a scientifically validated and personalized VR service to be used by older adults in their homes, which can improve their overall physical, cognitive, and social well-being.

**Objective:**

The main purpose of the CoSoPhy FX (Cognitive, Social, and Physical Effects) study was to analyze the effects of a VR-based digital therapeutics app on the cognitive, social, and physical performance abilities of healthy (high-functioning) older adults. This paper presents the study protocol and the results from the recruitment phase.

**Methods:**

A group of 188 healthy older adults aged 65-85 years, recruited at the Medical University of Lodz, Poland, were randomly allocated to the experimental group (VR dual-task training program) or to the control group (using a VR headset app showing nature videos). A total of 3 cognitive exercises were performed in various 360° nature environments delivered via a VR head-mounted display; the participants listened to their preferred music genre. Each patient received 3 sessions of 12 minutes per week for 12 weeks, totaling a minimum of 36 sessions per participant. Attention and working memory (Central Nervous System Vital Signs computerized cognitive battery) were used as primary outcomes, while other cognitive domains in the Central Nervous System Vital Signs battery, quality of life (World Health Organization–5 Well-Being Index), health-related quality of life (EQ-5D-5L), and anxiety (General Anxiety Disorder 7-item questionnaire) were the secondary outcomes. The group-by-time interaction was determined using linear mixed models with participants’ individual slopes.

**Results:**

In total, 122 (39%) of the initial 310 participants failed to meet the inclusion criteria, resulting in a recruitment rate of 61% (188/310). Among the participants, 68 successfully completed the intervention and 62 completed the control treatment. The data are currently being analyzed, and we plan to publish the results by the end of September 2024.

**Conclusions:**

VR interventions have significant potential among healthy older individuals. VR can address various aspects of well-being by stimulating cognitive functions, promoting physical activity, and facilitating social interaction. However, challenges such as physical discomfort, technology acceptance, safety concerns, and cost must be considered when implementing them for older adults. Further research is needed to determine the long-term effects of VR-based interventions, optimal intervention designs, and the specific populations that would benefit most.

**Trial Registration:**

ClinicalTrials.gov NCT05369897; https://clinicaltrials.gov/study/NCT05369897

**International Registered Report Identifier (IRRID):**

DERR1-10.2196/53261

## Introduction

### Background or Aims

The global demographic shift toward an aging population presents a pressing need to enhance the quality and effectiveness of health care [1]. Aging is typically associated with cognitive inactivity and, thus, cognitive impairment, resulting in escalating care demands and health care costs [2] and reducing daily functioning and quality of life among older adults [3]. As such, there is a need for effective interventions aimed at enhancing cognitive functions, which can mitigate their decline and promote healthy aging [4].

A promising tool with significant potential across various fields is virtual reality (VR), which has been found to be effective in caring for older individuals, both with and without cognitive impairment [5]. By offering immersive digital experiences, VR can provide varying levels of immersion and high immersion can be provided by head-mounted displays (HMDs), which can stimulate multiple sensory modalities [6]. Such heightened immersion enhances the sense of presence, yielding more pronounced behavioral responses [7].

In addition to the general cognitive function, VR-based training programs have shown promise in improving specific cognitive domains, such as short-term memory, orientation, attention, comprehension, naming, constructions, memory, and judgment [8]. Indeed, VR interventions have been found to significantly improve executive function and verbal memory in older adults [9], whereas traditional cognitive training methods may be limited in terms of accessibility, motivation, and ecological validity [10]. VR technology offers a unique opportunity to create immersive and engaging 3D experiences that simulate real-world scenarios and involve multiple sensory modalities [11,12]. Indeed, studies indicate that VR interventions can enhance the effectiveness and engagement of cognitive interventions, resulting in better outcomes among older patients [13]. VR-based training may also have a beneficial influence on physical activity, with research suggesting it can improve balance performance and functional mobility [14]. Additionally, the potential of VR interventions to promote social engagement and combat social isolation has also been studied among older adults [15].

Research has shown that music has many positive effects on human health, including reducing stress and anxiety [16], enhancing mood [7], improving sleep [17], and relieving pain [18], and it can be an excellent motivator for physical activity [19]. Music has previously been integrated into VR experiences among older adults [11] to increase motivation [11], improve mental well-being [20], and support rehabilitation [21].

While studies have examined the use of VR in health care settings, particularly in mental health [22], its application in healthy older adults who can use VR at home is still an emerging area of research [11]. Understanding the benefits and potential limitations of home-based VR interventions is crucial for developing evidence-based strategies to support healthy aging [4]. Further research is still needed to assess the impact of VR interventions on specific cognitive, physical, and social domains and to identify the optimal protocols for therapeutic use.

Currently, existing VR products and services are prohibitively expensive for many home end users, and their use is typically limited to clinics, nursing homes, and rehabilitation centers [23]. However, the most effective strategy for ensuring maintainable health care in the face of aging populations is prevention. As such, there is a need to provide a home-based service that offers scientifically validated, safe, engaging, and personalized content for older adults to enhance their cognitive, physical, and social abilities.

### Objective

The main aim of the study is to determine the effects of using a VR-based digital therapeutics app to improve the cognitive, social, and physical performance abilities of healthy (high-functioning) older adults.

### Experimental Hypotheses

It is expected that significant relationships will be found between the 2 factors (group × time) for each of the tested cognitive, physical, and social performance scores. Specifically, cognitive, social, and physical performance scores are expected to significantly improve throughout the VR training intervention compared with the start, but only for the experimental group. No such change is expected to be observed for the control group.

## Methods

### Trial Design

The Cognitive, Social, and Physical Effects (CoSoPhy FX) study was designed as a randomized, parallel-group, 2-arm, superiority study with an aimed 1:1 allocation ratio. The protocol was registered with ClinicalTrials.gov (NCT05369897) on June 30, 2022.

### Participants

#### Recruitment

In total, 200 high-functioning older adults aged 65-85 years from a community-dwelling setting were recruited for the study. All recruitment was performed by the Medical University of Lodz (MUL), Poland. High-functioning older adults were defined as those who were older than 65 years of age and maintained their functional independence concerning activities of daily living, including the ability to go on a long walk and to interact with standard modern technology (eg, using a smartphone to send a message).

The recruitment strategy included the following: (1) promotional materials such as posters, flyers, press announcements, and posts on social media groups devoted to older adults were used to raise awareness in the local community and inform older adults about the project; (2) a dedicated phone line operating during working hours was set up to answer inquiries regarding participation in the study; and (3) study visits were carefully planned and scheduled to work around holiday breaks.

The enrollment period lasted over 12 months. The allocated research team member screened the potential participants according to the eligibility criteria, given in Textbox 1.

**Inclusion and exclusion criteria.**

**Inclusion criteria**

- Individuals aged 65-85 years
- Stable medical condition (eg, well-controlled diabetes or hypertension)
- Undisturbed locomotion
- Independent in everyday functioning
- Capable of going on long walks without assistance
- Able to use standard modern technology

**Exclusion criteria**

- Neuropsychiatric disorders (Montreal Cognitive Assessment<26 points)
- Abuse or addiction to alcohol, drugs, and tranquilizers (*Diagnostic and Statistical Manual of Mental Disorders, Fifth Edition*)
- Blurred vision that cannot be corrected with lenses or glasses
- Auditory pathologies causing significant hearing loss
- High sensitivity to motion sickness
- Migraines
- Epilepsy
- Obesity (BMI>30)
- Deemed unsuitable for participation by the investigator

The clinical and demographic data that were collected during the screening were age, sex, educational level, marital status, socioeconomic status, data on chronic diseases, and medications. A clinical assessment was conducted using the Montreal Cognitive Assessment (MoCA) test [24,25] to exclude participants with suspected cognitive impairment (ie, achieving a total MoCA score lower than 26 points). After qualification, the participants were randomly allocated to either the experimental or the control group. These participants were invited for a second visit, during which they completed a battery of tests for neuropsychological and psychosocial assessment and were trained on how to use the VR-HMD on their own.

#### Randomization

After the participants had been screened for eligibility, the research team produced a list of eligible participants. Participants were randomly allocated to an experimental or control group with a 1:1 allocation using a computerized random number generator [26]. A simple randomization method was adopted. One author (ES) generated sequences of numbers from 1 to 200 and assigned them to the intervention based on the assumption that 1-100=experimental group and 101-200=control group. Another author (AA) assigned the participants to either the experimental or control group according to the list of randomized numbers. To ensure that the researchers were blinded, the list was kept by an independent research team member (JK) who did not participate in the participant recruitment process. No other stratification was used.

#### Ethical Considerations

The protocol and the informed consent form (Multimedia Appendix 1) received approval from the Bioethical Committee of the MUL, Poland (RNN/222/21/KE). Each participant was introduced to the study by a research team member, who performed an initial demonstration of the equipment. The participants were given time to ask any remaining questions. Following this, if they agreed to take part, they signed a form giving their written informed consent (Multimedia Appendix 1).

All data collected during the study will be entered into a secure, password-protected electronic database (internal cloud service and protected from external access). The data set for statistical analysis was fully anonymized. Participants’ paper files will be stored in numerical order and in a secure place and manner. A coding system will be developed to ensure uniformity and consistency in categorizing variables. Each variable will be assigned a unique code that will be used throughout the study for data entry and analysis. Access to the data will be restricted to authorized personnel only. Data will be stored on a secure server with regular backups to prevent data loss. Personally identifiable information will be stored separately from the research data to maintain participant confidentiality.

In the conduct of this research, all human participants participated voluntarily and without the promise or receipt of financial compensation or any other form of remuneration.

### Intervention

#### Overview

The intervention was delivered using a VR-HMD worn by the older adults—a Pico Neo 3 Pro (Pico Interactive) in the experimental group and a Pico G2 in the control group. The content shown in the VR-HMD consists of 2 key components.

#### Base Content

The base consent comprises high-quality 360° photographs and videos from natural environments in the real world, such as a relaxing environment with a mountain view. Initially, 80 different base content environments were used.

#### Graphical Overlay

##### Overview

The base content has a graphical overlay that has 2 key components.

##### Hands

The user’s hands and their position are shown inside the VR environment as virtual hands, moving in real time. This requires the user to hold a controller in each hand. The user’s hands and their positions are displayed within the VR environment as digital hands that move in real time. This setup requires the user to hold a controller in each hand.

##### Graphical Objects

Graphical objects are, for example, balloons, that users can touch using their virtual hands. These appear inside the VR environment and move in prespecified trajectories. Graphical objects, such as balloons, can be touched by users through their digital hands. These objects appear in the VR environment and follow predetermined trajectories. The intentions of this design were (1) to perform physical exercises (moving the arms and upper body to touch the graphical objects) and (2) to perform cognitive exercises (using the arms to touch graphical objects according to specific rules for each cognitive task).

**Figure 1:**
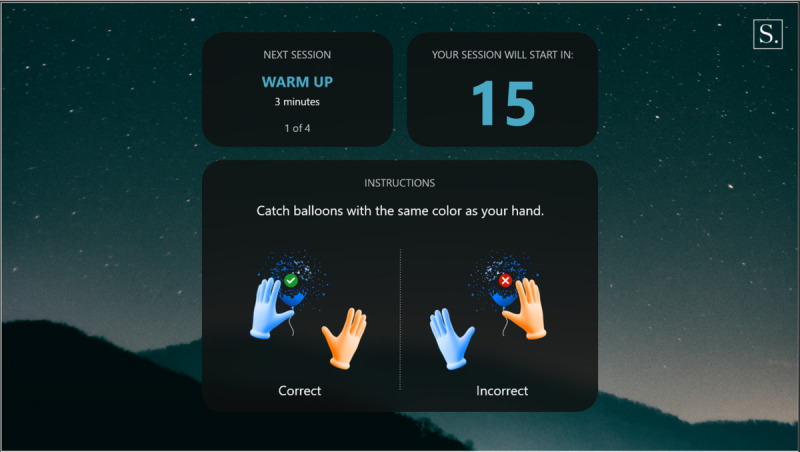
Warm-up introduction.

**Figure 2:**
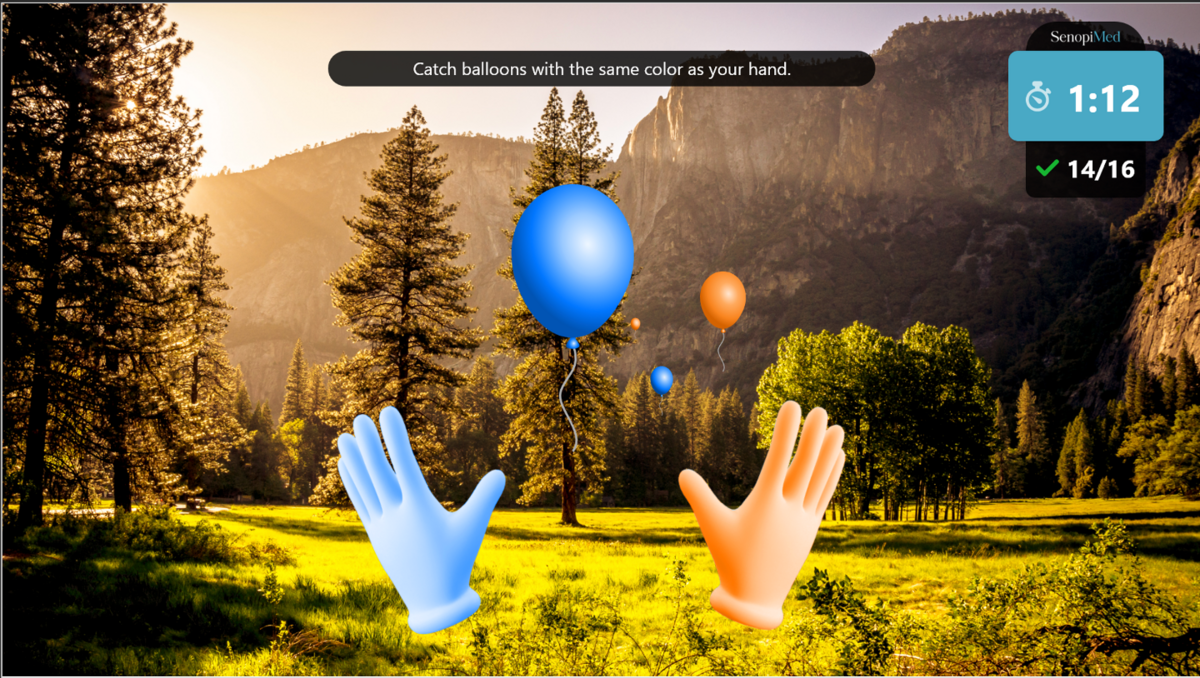
Warm-up exercise.

**Figure 3:**
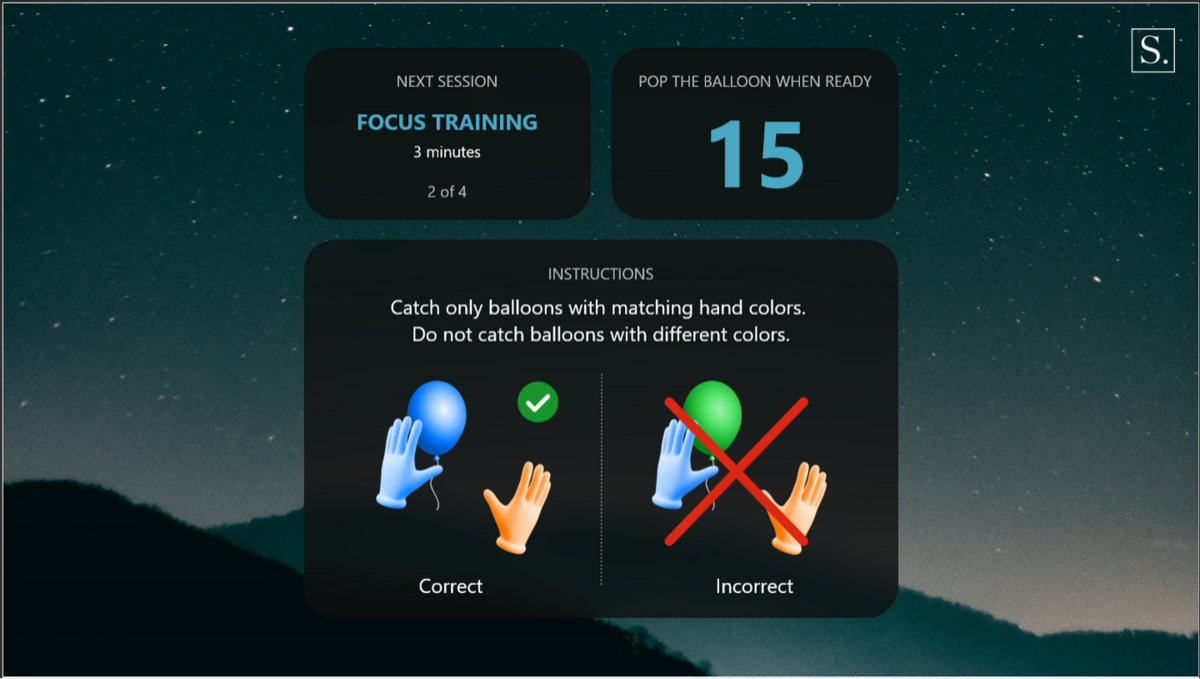
Focus introduction.

**Figure 4:**
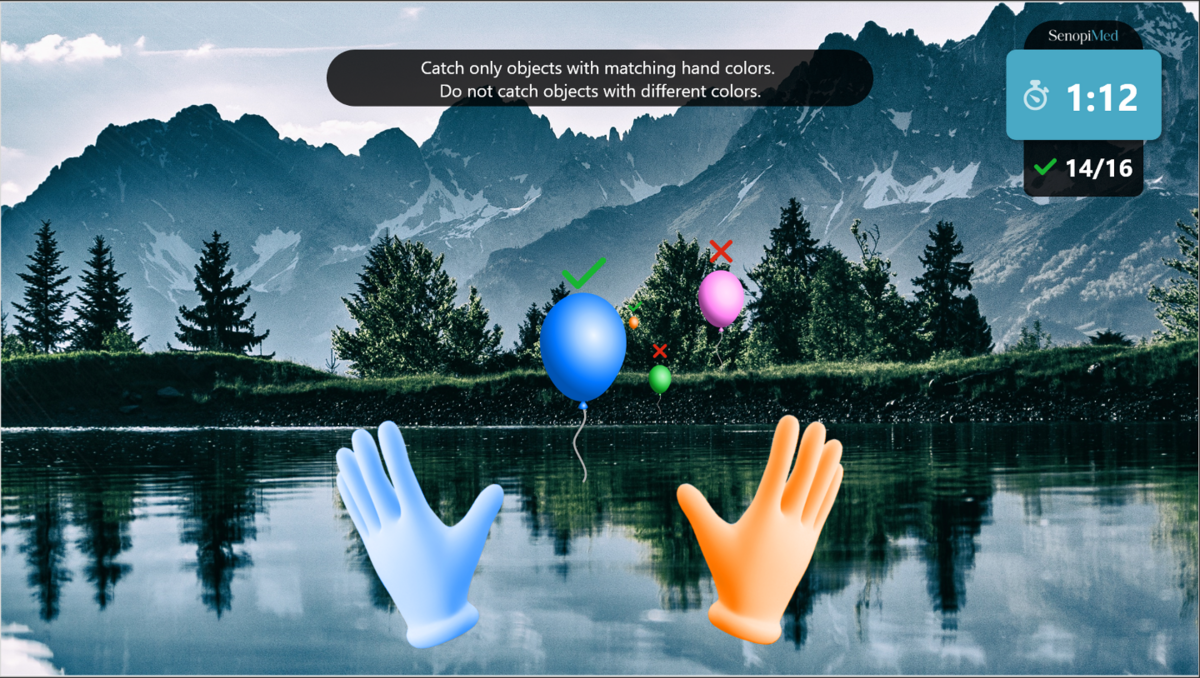
Focus exercise.

**Figure 5:**
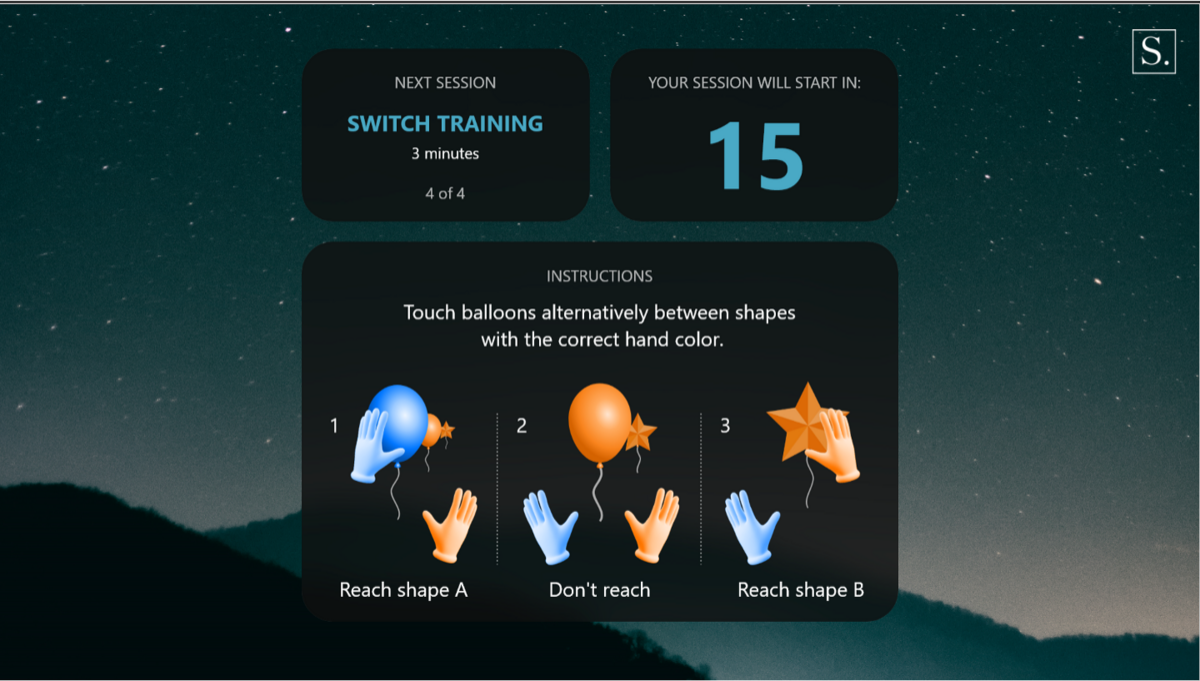
Switch introduction.

**Figure 6:**
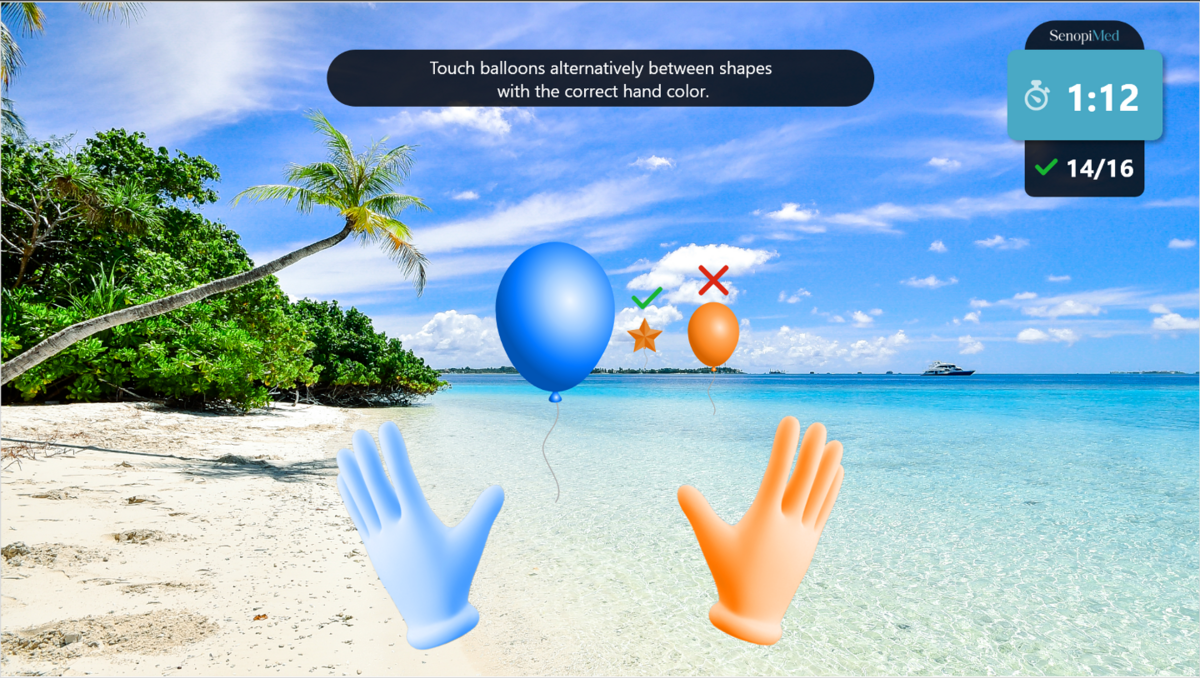
Switch exercise.

**Figure 7:**
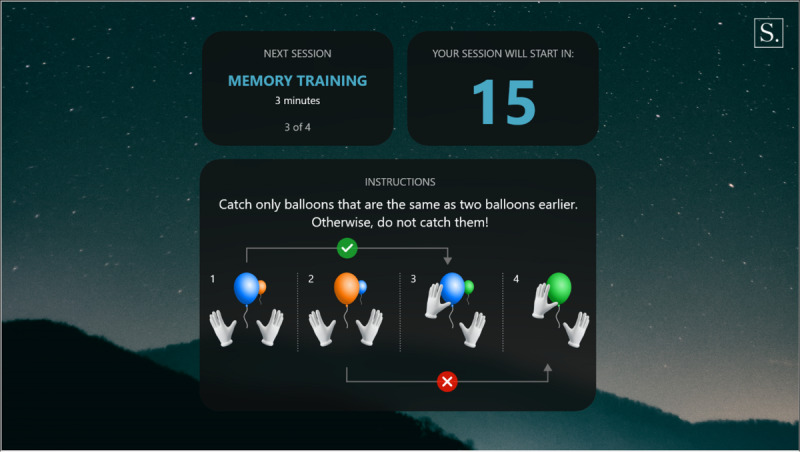
Memory introduction.

**Figure 8:**
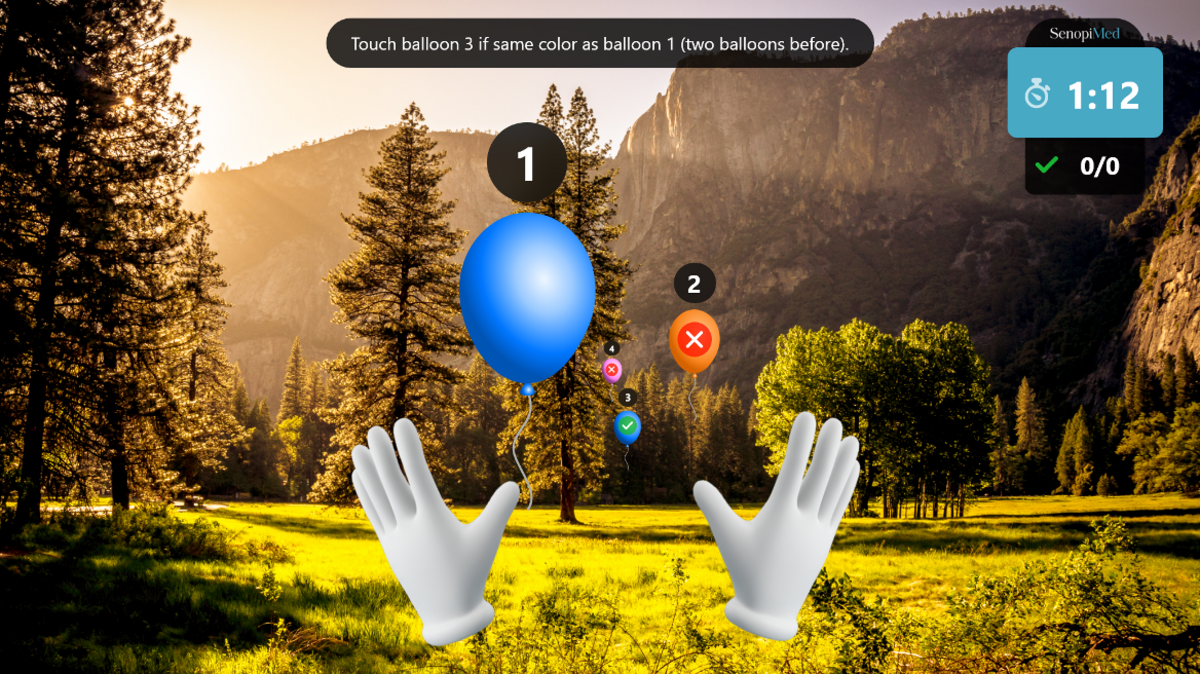
Memory exercise.

While performing the physical and cognitive exercises, the participants could also listen to a selection of musical pieces from the HealthTunes database. The music was played in stereo (48 kHz 24-bit WAV files, –15 dB LUFS) on the Pico G2 and Pico Neo 3 Pro VR-HMDs. All tracks were organized by HealthTunes [27]; none had any drastic tempo changes that could adversely entrain participants or any drastic dynamic changes that could interfere with the experience. The levels of the audio frequency bands were also adjusted to optimize the music to the older adults’ hearing. The selection of musical pieces is presented in Multimedia Appendix 2. Participants were provided with a choice of music spanning 4 genres—classical, ambient, electronic, and jazz, with the option to select rock music. This diverse selection was intended to accommodate individual preferences and enhance the personalization of the VR experience. Classical music was the most popular choice among participants, featuring a variety of operas and symphonies or concerti from renowned composers, offering a rich tapestry of auditory stimuli conducive to cognitive stimulation and relaxation. The cognitive exercises that were implemented within the VR app are as follows:

1. Warm-up: a simple exercise to familiarize the participant with each task, in which participants must select the graphical objects that match the color of their hands. The warm-up introduction is presented in Figure 1 and a warm-up exercise is presented in Figure 2.
2. Focus: an exercise of focused attention in which participants must select the graphical objects that match the color of their hands and avoid those that do not. The focus introduction is presented in Figure 3 and a focus exercise is presented in Figure 4.
3. Switch: an exercise for alternating attention in which participants must reach alternately for the shapes of the graphical objects with the matching hand color. The switch introduction is presented in Figure 5 and a switch exercise is presented in Figure 6.
4. Memory: an exercise of working memory based on the n-back task, in which participants must tap a graphical object if it is the same color as the one that appeared 2 graphical objects earlier. The memory introduction is presented in Figure 7 and the memory exercise is presented in Figure 8.

The targeted sample size was 200 participants, equally divided between the experimental and the control groups. Both groups were balanced regarding sex.

The experimental group members underwent a 3-month computerized VR dual-task training program, as described. The control group passively experienced 360° photographs and videos from natural environments without dual-task physical and cognitive training. This technique is frequently used in cognitive studies, offering a passive experience devoid of cognitive challenges [28]. In both groups, the participants were encouraged to use the VR-HMD for a minimum of 3 sessions of 12 minutes per week for 3 months, that is, for at least 36 sessions in total.

The first training session occurred at the MUL, where the participants were trained on how to use the VR-HMD. At the end of the session, the participants took the VR-HMD to their homes to use them according to the schedule described.

The adverse effects of using VR headsets, often referred to as cybersickness, have been reported to include eye strain, dizziness, nausea, and vomiting [29]. However, these side effects are most often due to suboptimal use of the technology, for example, by using VR content with quick changes of position, low image resolution, and low display refresh rates. The VR app used in the study was designed specifically for older adults and avoided any such drastic camera movements.

The app used in the control group had previously been tested by older adults in 15 older adult care homes in Switzerland and Finland. The first version of the app, containing cognitive training, used in the experimental group was tested by 30 older adults in usability studies by terzStiftung in Switzerland between September 2021 and January 2022. After 8 workshops, the feedback was used to develop updated versions of the app. The resulting cognitive training program, image quality, and usability were rated as high by the participants [30]. The results confirm that VR-HMDs are safe for use by high-functioning healthy older adults at home without substantial risks or side effects.

Nevertheless, participants received explicit instructions to discontinue the VR intervention and contact the researchers if they experienced any side effects. Moreover, in the case of suspected side effects, the participants would be withdrawn from the study, and this would be reported as an adverse event. Any side effects could be reported through a software app running on their mobile phone, tablet, or computer. At the end of the intervention, an open-ended questionnaire was used to collect feedback, including information on perceived side effects.

To acquire reliable and comprehensive outcome data, strategies were used to promote participant retention and ensure follow-up completion (Textbox 2). If participants discontinued the intervention or deviated from the protocol, information regarding their decision was collected with various self-reported outcome data related to their experience, expectations, perceived benefits, and any changes observed during their participation.

**Promoting participant retention and ensuring follow-up completion.**

**Clear expectations**

- The study requirements, including the duration of the intervention, the frequency of sessions, and any follow-up assessments, will be communicated. A comprehensive overview of the study timeline should be provided to participants, and their continued participation should be emphasized.

**The informed consent process**

- During the informed consent process, efforts will be made to ensure that participants fully understand the study’s goals, procedures, and potential benefits. Concerns expressed by participants will be addressed and their questions will be answered to foster their commitment to the study.

**Flexible scheduling**

- Participants’ schedules and preferences will be considered when planning pre- and postintervention assessments. Flexibility in scheduling will be offered to accommodate their availability, facilitating the completion of the required sessions.

**Reminders and communication**

- Periodic reminders about upcoming sessions or assessments will be sent to participants via SMS text messages and phone calls. Clear instructions, directions, and any necessary materials or equipment for the virtual reality –based training will be provided to participants.

**Supportive staff**

- A dedicated research team that is easily accessible to participants will be established. Staff members will be trained to provide emotional support and address participants’ questions or concerns during the study.

Compliance and performance were monitored through a web-based platform. The researchers contacted participants who did not perform the VR training at least 3 times per week to check whether they faced difficulties using the VR app or wished to discontinue participation. Throughout the intervention, the participants were expected to avoid participating in any other trials, especially those related to cognitive functions or mental states. The participants were covered by health insurance throughout their participation in the study, concerning any potential harm, according to a standard clinical study insurance policy.

### Outcome Measures

Neuropsychological assessment was performed at baseline and after the intervention. The primary outcomes of the initial study comprised the cognitive, social, and physical performance abilities of healthy (high-functioning) older adults. The parameters that were selected are (1) a wide range of cognitive domains: composite memory, verbal memory, visual memory, psychomotor speed, reaction time, complex attention, cognitive flexibility, processing speed, executive function, simple attention, and motor speed (Central Nervous System Vital Signs [CNS-VS] battery) [31]; (2) physical performance across 6 subcomponents: lower body strength, upper body strength, aerobic endurance, lower body flexibility, upper body flexibility, and dynamic balance or agility (Senior Fitness Test); (3) standard grip strength (using a dynamometer); (4) the sense of loneliness (De Jong Gierveld Loneliness Short Form); (5) the quality of life and perceived well-being (World Health Organization [WHO]–5 Well-Being Index); (6) the health-related quality of life (EQ-5D-5L); (7) satisfaction with participation in social roles and ability to participate in social roles and activities (Patient-Reported Outcomes Measurement Information System Short Form 8a); and (8) the severity of anxiety symptoms (General Anxiety Disorder 7-item questionnaire [GAD-7]) [32].

The CNS-VS is a computerized neurocognitive test battery developed as a routine clinical screening instrument. It comprises 7 tests—verbal and visual memory, finger tapping, symbol digit coding, the Stroop Test, a test of shifting attention, and the continuous performance test. The result includes neurocognitive clinical evaluation domains—composite memory, verbal memory, visual memory, psychomotor speed, reaction time, complex attention, cognitive flexibility, processing speed, executive function, simple attention, and motor speed [31]. The CNS-VS clinical battery is sensitive to subtle cognitive deficits and progressive decline or improvement. Previous research has shown it is validated, reliable, and easy to use [33].

The WHO-5 Well-Being Index is a 5-item measure of well-being with high internal consistency, evidence of a 1D factor structure, and high convergent associations with other measures of well-being [34,35]. The respondent is asked about their well-being in the last 14 days. Each of the 5 items is scored from 5 (all of the time) to 0 (none of the time). The raw score ranges from 0 (absence of well-being) to 25 (maximal well-being) [36].

The EQ-5D-5L is a questionnaire used to assess health-related quality of life. It measures the problems experienced in 5 domains, which are, mobility, self-care, usual activities, pain or discomfort, and anxiety or depression. The questionnaire also includes an overall health scale where individuals rate their health condition from 1 to 100, with 100 representing the best imaginable health. Previous research has found the EQ-5D-5L to be reliable and valid [37].

The GAD-7 was used to measure the severity of anxiety symptoms. Each item considers different anxiety symptoms and is answered on a 4-point Likert-like scale. The total score ranges from 0 to 21, and a rise in the score indicates more severe anxiety. The tool is sensitive to anxiety in nonclinical samples and to changes in the severity of anxiety symptoms.

Grip strength was assessed with a hand-held dynamometer. The method is a reliable measure of the strength of muscles in older adults when using a standardized protocol and instructions [38]. The resulting grip strength data have been reported to predict general muscle strength and physical functioning in the older adult population [39,40].

### Challenges

On September 21, 2021, KB, SS, JK, LM, ES, KN, MMH, and WW prepared the original version of the protocol (version 1.0). During the further development of the VR-based digital therapeutics app, it was decided to eliminate the physical and social aspects of the intervention. On January 10, 2022, the protocol concerning outcome measures was amended (version 2.0); the primary outcomes were redefined as attention and working memory. Secondary outcomes comprised other cognitive domains, quality of life, well-being, anxiety, and standard grip strength. The same authors were involved in making the amendment, and all the changes to the protocol and outcomes were made before participant recruitment. All relevant parties (including the institutional review board and investigators) were informed about the protocol amendments and outcomes. This decision was prompted by several limitations experienced when using the solution at home, such as insufficiently fast internet, which was needed for trouble-free operation, difficulties in connecting to Wi-Fi, a complex setup process, and the risk of falling.

### Statistical Methodology

An a priori computation of the required sample size based on the type of test, the expected effect size, the type I and type II error rates, the number of groups, and the number of measurements were performed according to Lu et al [41]. The statistical power analysis was conducted for a mixed model of repeated measures for 2 groups, 1 measure at a time (corresponding to the main cognitive performance scores detailed) with a 0.7 pre-post correlation, a 20% dropout rate, a medium effect size (0.5), a significance level of α=.05, and a statistical power of 1–β=.80. The necessary sample size for these parameters was 77 (n=154) participants per group.

The MUL stored the personal data of the interested and eligible participants on an internal cloud service, protected from external access. The data set for statistical analysis was fully anonymized. All data storage and processing comply with the General Data Protection Regulation (GDPR) and other applicable regulations. Where possible, fully anonymized data may be made publicly available through open-access repositories supporting the Open Science Initiative.

## Results

### Participant Flow

**Figure 9:**
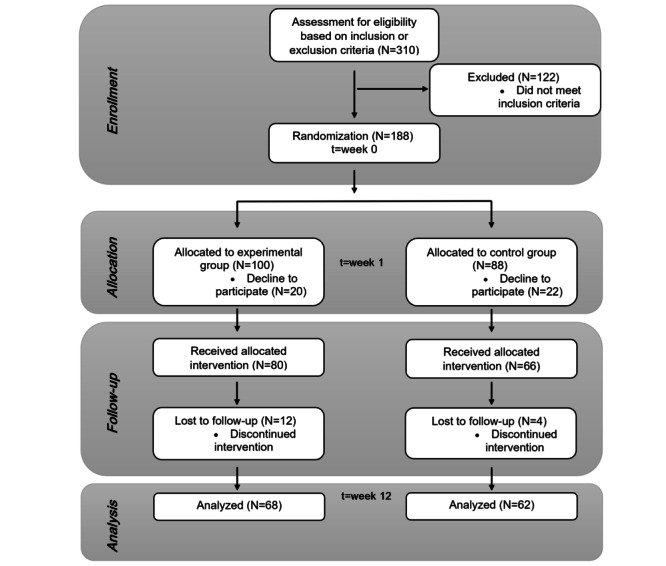
CoSoPhy FX (Cognitive, Social, and Physical Effects) study flowchart. Study design: a randomized, parallel-group, 2-arm, superiority study; allocation ratio: 1:0.88; population: individuals aged 65-85 years; location: Poland; time frame: 3 months.

The project received funds from June 1, 2021, to October 31, 2023. The study’s design, management, analysis, and reporting are independent of the funder. The study, from recruitment to completion of follow-up, was planned to take place from January 2022 to May 2023. The recruitment phase was performed from January 2022 to mid-January 2023. As shown in Figure 9, a total of 310 participants were tested for eligibility and 122 were excluded because of not meeting the inclusion criteria. The most common cause of exclusion was the MoCA score being below 26 points, suggesting the presence of objective cognitive impairment. The recruitment rate (61%, 188/310) was acceptable. Following this, 188 participants were allocated to an experimental group (n=100, 53.2%) or a control group (n=88, 46.8%), with an allocation ratio of 1:0.88.

In the experimental group, 20 participants declined to participate before the start of the intervention and withdrew their consent without giving a reason. A total of 12 participants withdrew because they reported somatic and mental health problems unrelated to the intervention. In the experimental group, 68 participants completed the intervention.

In the control group, 22 participants declined to participate before the start of the intervention and withdrew their consent without giving a reason. A total of 4 participants withdrew because they reported somatic and mental health problems unrelated to the intervention. In the control group, 62 participants completed the intervention. There were no missing data.

The data are currently being analyzed, and we plan to publish the results by the end of September 2024.

### Dissemination

The study results will be published in scientific journals and presented at appropriate scientific conferences. All materials and papers will be written by the researchers. No services of external copywriters will be used. The study protocol was registered and published on ClinicalTrials.gov.

## Discussion

### Potential Benefits

VR-based interventions can significantly improve the quality of life for healthy older individuals in several ways. First, VR offers a means of mental stimulation and cognitive engagement. By immersing the user in various environments, tasks, and games, VR can delay cognitive decline by enhancing various cognitive abilities such as memory, attention, and problem-solving skills [42,43]. Second, VR interventions can be used to support physical activity and rehabilitation by providing a safe environment for older adults to improve their balance and flexibility and reduce the risk of falls and injuries [14]. Third, VR provides opportunities for social interaction and connectivity, thus combating the isolation and loneliness often experienced by older people. VR meetings and games can promote engagement with family and peers, and exploring VR worlds can offer a sense of adventure and travel without physical limitations [15]. Finally, VR apps can be used to manage anxiety and stress, promote general well-being, and provide leisure opportunities, thus positively impacting quality of life [44].

### Potential Risks and Challenges

Several potential risks and challenges are associated with using VR interventions in the older adult population, and these should be taken into consideration to maximize their benefits and minimize any potential harm. First, suboptimal and prolonged use may be associated with physical discomfort or cybersickness, comprising symptoms such as nausea, disorientation, or eyestrain [29]. Second, there can be a significant learning curve for older adults unfamiliar with the technology, and some individuals may find the technology intimidating or stressful, leading to rejection or noncompliance [45]. Third, if the person is unaware of their real-world surroundings while immersed in the VR environment, they may be at risk of falls or accidents. Finally, while the cost of VR hardware has decreased, it can still be expensive, especially for high-quality systems.

### Limitations

One limitation of this study is that it did not determine the degree of function of the participants by an appropriate objective tool; instead, it was evaluated by a research team based on a medical interview during the screening process. While this approach provided some insight into the participants’ functional independence in activities of daily living, it may have introduced subjectivity and potential bias. Objective tools, such as standardized assessments or performance-based measures, could have provided more reliable and valid measures of older adults’ functioning. For instance, widely recognized tools such as the Timed Up and Go (TUG) [46] test for mobility and the Instrumental Activities of Daily Living (IADL) scale [47] for more complex tasks could offer standardized and quantifiable metrics. Future studies should consider incorporating these objective tools to obtain a more comprehensive and accurate assessment of functional ability.

Another potential limitation of the study relates to the occurrence of cybersickness among participants engaging with VR interventions. Cybersickness, a condition characterized by symptoms such as dizziness, nausea, and headache, mirrors the symptoms of motion sickness but is induced by immersive VR environments. The incidence of cybersickness varies among individuals and can significantly impact the user’s ability to engage with VR-based interventions over time [29]. This limitation is particularly relevant as it may affect the participant’s adherence to the intervention, potentially skewing the study’s outcomes. Future research should aim to identify strategies to minimize cybersickness, possibly through the adaptation of VR content, the duration of sessions, or the inclusion of breaks, to enhance the comfort and participation of all users.

Furthermore, the study faced challenges associated with Wi-Fi connectivity, which represents another significant limitation. A reliable Wi-Fi connection is crucial for the seamless delivery of VR content and the uninterrupted participation of subjects in the study. Issues with Wi-Fi connectivity, such as signal instability or bandwidth limitations, can disrupt the VR experience, leading to fragmented sessions or the inability of participants to complete the intervention as planned. This technological constraint not only affects the consistency and quality of the intervention delivery but also potentially impacts the engagement and satisfaction of participants. Addressing Wi-Fi connectivity issues is essential for ensuring the fidelity of VR-based interventions, and future studies may need to consider alternative solutions or backup options to mitigate these challenges, ensuring a smooth and effective implementation of VR technology in research settings.

### Conclusions

Previous research found that VR therapy can improve the well-being of older adults in long-term care, rehabilitation hospitals, and older adult care residences, who often experience cognitive decline, reduced mobility, and isolation [22]. We intend to publish our findings in a future study, and we believe that they will provide further information on the effect of immersive VR therapy among healthy, community-dwelling older adults regarding their cognitive performance, tolerance, and technology acceptance. Even so, further research is needed to explore the long-term effects, optimal intervention designs, and specific populations that may benefit most from VR-based interventions.

## Appendix

Multimedia Appendix 1 Informed consent.

Multimedia Appendix 2 Music selection.

## Abbreviations

CNS-VS: Central Nervous System Vital Signs
CoSoPhy FX: Cognitive, Social, and Physical Effects
GAD-7: General Anxiety Disorder 7-item questionnaire
GDPR: General Data Protection Regulation
HMD: head-mounted display
IADL: Instrumental Activities of Daily Living
MoCA: Montreal Cognitive Assessment
MUL: Medical University of Lodz
TUG: Timed Up and Go
VR: virtual reality
WHO: World Health Organization

## Data Availability

The data sets generated and analyzed during this study are available from the corresponding author on reasonable request.
