# Supplementary material for: The Effectiveness of Virtual Reality–Based Training on Cognitive, Social, and Physical Functioning in High-Functioning Older Adults (CoSoPhy FX): 2-Arm, Parallel-Group Randomized Controlled Trial
Source: JMIR Res Protoc. 2024 Jun 5;13:e53261. doi: 10.2196/53261 (PMC11187518; doi:10.2196/53261)
Supplement: Multimedia Appendix 1 [file resprot_v13i1e53261_app1.docx]

**Informed consent form**

For participation in the project entitled: **CoSoPhy FX- *Bringing the beneficial cognitive, social and physical effects of immersive training to the high-functioning senior home-user***

I acknowledge that:

- I have read information for the participant;
- I understand all the information, including benefits and adverse effects;
- I have had sufficient time to think about my participation; and
- I have been able to ask questions. To the extent that I asked questions, they were answered satisfactorily.

Consciously and voluntarily, I agree to participate in the project described in the information for the participant.

I know that my participation is voluntary and that I may withdraw my consent at any time without giving a reason and without affecting the medical care I will be entitled to receive.

I accept the insurance conditions in connection with participation in the project.

I agree to the processing of my personal data in this project in accordance with the law in force in Poland and consent to the use of the collected data for scientific purposes. Survey, testing and performance data may be shared internationally with members of the project consortium in accordance with the purpose of the project, as well as with members of ethics committees and higher authorities.

I hereby guarantee that if I experience any adverse effects while using the VR application, such as eye strain, dizziness, nausea and vomiting, I will report this to the investigators.

First and last name of participant: .........................................................................................

Place, date, signature: ............................................................................................................

**CoSoPhy FX (*Bringing the beneficial cognitive, social and physical effects of immersive training to the high-functioning senior home user*)**

**Information for participant**

Ageing results in progressively decreasing abilities and quality of life. Seniors have limited access to stimulating activities, like nature excursions, visits to their families' homes, attending concerts, visiting museums, and travelling to tourist attractions. This limitation is exacerbated by the recent measures that have been put into effect to contain the spread of the Covid-19 pandemic, which have placed further restrictions on exercise, excursions, and social visits. There is a considerable likelihood that these measures will stay in effect or be reinstated periodically in response to future waves of Covid-19 or other pandemics. Furthermore, there is a notable lack of solutions addressing the needs of high-functioning seniors, who represent an exponentially growing demographic group.

The study evaluates whether training with a virtual reality (VR) system can significantly improve seniors' cognitive functioning and quality of life.

Your participation in the project will allow you to take advantage of a modern system based on VR, which aims to help you train your cognitive and motor abilities at home, thus contributing to improving your physical, mental, and social well-being. The proposed system allows for staying cognitively and physically active for longer, having a healthier lifestyle, and reducing the burden on families and organizations that provide care for older adults.

The operating principles and mechanism by which the system works are:

- the users sit comfortably in a chair with the knees at a 90-degrees angle and the back straight.
- the users wear a VR headset (head-mounted display) and turn it on.
- the users experience the VR application.

People aged 65-85 with undisturbed locomotion will be eligible for the project. You cannot take part in the study if you:

- do not agree to the examination;
- have not signed the informed consent form;
- have a psychiatric disorder;
- have a blurred vision that cannot be corrected with lenses or glasses;
- have an auditory pathology causing a significant decrease in hearing unaided;
- have a high sensitivity to motion sickness;
- are prone to migraines;
- have epilepsy;
- are vulnerable, abused, or addicted to alcohol, drugs, or sedatives.

As part of the project, the doctor will conduct an examination during which the following tools will be used:

- Montreal Cognitive Assessment;
- CNS Vital Signs battery and its subcomponents;
- GAD-7
- WHO 5 Well-Being Index;
- EQ5D5L
- a standard grip strength measurement using a dynamometer;

Testing primary and secondary outcomes will be performed at two longitudinal time points:

1. before the intervention (as baseline reference performance)
2. after three months of regular use of the VR application.

Two hundred healthy seniors will use one of two VR applications for at least 12 minutes at least three days per week for three months. Each participant will be randomly allocated to the experimental or control group.

The doctor performing the assessment will inform you about the examination results during the visit after three months of VR training. Participation in the study is voluntary and does not involve any risk for participants. However, following the 'Instructions for using the equipment' is essential to ensure safe usage. It will be possible to withdraw from the project at any time during the study without giving any reason or consequences. Documentation enabling the participant's identification is confidential, and personal data is excluded from any study description. In addition, you will receive your copy of the present document. You can ask questions at any time that should result in satisfactory answers. You will be insured against any potential damages incurred as a result of participating in the project. The collected data will be protected in accordance with medical confidentiality and the Personal Data Protection Act.

If you experience eye strain, dizziness, nausea, vomiting, or any other adverse effect while using the VR application, this should be reported to the investigators using the telephone numbers below.

Contact: [names and telephone numbers blinded]
